# MagNanoTrap enrichment empowers ultra-sensitive quantification of mixed nanoplastic particles from environmental water samples

**DOI:** 10.21203/rs.3.rs-6254645/v1

**Published:** 2025-07-14

**Authors:** Maochao Mao, Marian Bienstein, Francisca Contreras, Dong Wang, Lilin Feng, Ulrich Schwaneberg

**Affiliations:** 1 Lehrstuhl für Biotechnologie, RWTH Aachen University, Worringerweg 3, 52074 Aachen, Germany

## Abstract

Detection and quantification of nanoplastic particles (NPs) in environmental water are important for monitoring NPs’ fate and assessing health impacts, but the lack of sensitive and universal detection systems hinders regulation according to the EU Commission (Allan, J. et al., 2021). The MagNanoTrap termed enrichment platform is based on a bifunctional peptide (LCI-DZ-MBP1) combined with Fe_3_O_4_ superparamagnetic nanoparticles (SPIONs); the bifunctional peptide was designed to decorate SPIONs for NPs capture, with the LCI-peptide binding to SPIONs and the MBP1-peptide acting as a general binder for polypropylene (PP), polyethylene (PE), polystyrene (PS), and polyethylene terephthalate (PET) particles. The MagNanoTrap enrichment platform, with a maximum MagNanoTrap adsorption capacity of 3.95 ± 0.14 g/g for PS-COOH _500 nm_ NPs, enables in combination with pyrolysis-gas chromatography/mass spectrometry (Py-GC/MS) to reliably determine the composition of mixed NPs and quantify NP amounts down to 0.061 µg for PS. The achieved sensitivity ensures that a 1 L water sample is usually sufficient to detect and quantify NPs in environmental water samples, requiring only 16 mg of LCI-DZ-MBP1 coated SPIONs. The high affinity and general applicability of MagNanoTrap against NPs are ensured through high salt ion concentration, which makes hydrophobic interactions the main binding force. Proof of concept for versatile use of the MagNanoTrap enrichment platform was successfully performed by enrichment and quantification of mixed NPs, including PP, PS, PE, PET, poly (methyl methacrylate), polycarbonate, nylon 6, and nylon 66, across seven types of environmental water samples from rivers, lake, sea, and wastewater sources.

## Introduction

Polymers such as polypropylene (PP), polystyrene (PS), polyethylene (PE), polyethylene terephthalate (PET), poly (methyl methacrylate) (PMMA), polycarbonate (PC), nylon 6, and nylon 66 were produced in 413.8 million metric tons in 2023 (Plastics – the fast Facts 2024 • Plastics Europe), due to their excellent properties in mechanical strength, ductility, and corrosion resistance, etc. A responsible use of polymers requires the development of a sustainable circular polymer economy^1^. Policies, such as limits on virgin plastic production, reductions in unnecessary plastic use, and investments in waste and recycling management will very likely lead to significant progress in both open- and closed-loop polymer recycling^2, 3^. However, in a circular polymer economy, one can expect that the number of nanoparticles in the environment will dramatically increase since all kinds of materials will be either biodegradable or recycled^4^. For instance, out of 1 g of polyester textiles, up to 100 billion plastic nanoparticles within the size of 1 µm are released^5, 6^. Nowadays, micro-/nanoplastics (MP/NPs) are globally pervasive, contaminating environments from the atmosphere^7, 8^ to water systems like rivers^9, 10^, lakes^9, 11^, and oceans^12^, and can be even found in food (e.g., honey^13^ and infant formula^14^) as well as bottled water^15^ or beverages^16^. The four most commonly reported MP/NP contaminations are PP, PE, PS, and PET^17^, with hydrophobic polymers particularly known for accumulating toxic hydrophobic compounds, such as polycyclic aromatic hydrocarbon (PAHs), plastic additives, and pharmaceuticals^18^. Recent studies have linked the accumulation of MP/NPs in plant roots to inhibit plant growth and seed germination^19–21^. The ingestion of MP/NPs by animals has also been reported^22, 23^, with mussels^24, 25^, and zebrafish^26^ being proposed as biomarkers for MP/NP contaminations^25^. Furthermore, reports indicate the accumulation of MP/NPs in human brians^27^ and particularly, NPs below 20 nm could cross the blood-brain barrier, contributing to neurological disorders (e.g., amyotrophic lateral sclerosis^28^ and Parkinson’s disease^29^).

In essence, to ensure environmental and human health, it is important to develop enrichment methodologies to identify and quantify MP/NPs in aqueous environments, beverages, and food products^4^. The analytical gaps to reliably quantify particles below a size of 5 mm have been acknowledged by the EU Commission as a main limitation in introducing regulations on MP/NP contaminations^30, 31^. To date, reported remediation technologies for the removal of MP/NPs comprise flotation systems based on bubble-particle interactions^32, 33^, membrane-based approaches leveraging the benefits of membrane cut-off^34–38^, and physically or chemically surface-functionalized materials driven by adsorption process^39–42^. However, only membrane-based approaches have been reported as generally applicable for monitoring MP/NP contamination in environmental water samples. Specifically, these approaches have been implemented in cross-flow systems requiring 50 L of environmental water samples^35, 36^ and dead-end systems processing 1 L samples^36–38^. Both systems utilized Polyethersulfone (PES) membranes with a 0.01 µm cutoff and were coupled with pyrolysis-gas chromatography/mass spectrometry (Py-GC/MS) for quantifying mixed plastics, including PP, PE, PS, PET, PMMA, PC, Nylon 6, and Nylon 66, achieving recovery exceeding 50%^35–38^. Surface-functionalized magnetic adsorbents, commonly based on Fe2O3/Fe3O4 that are often referred to as superparamagnetic iron oxide nanoparticles (SPIONs), comprise magnetic micro-/nanorobots^43–45^, magnetic metal-organic frameworks (MOFs)^46, 47^, and chemically-modified magnetic beads^48, 49^. These systems have shown removal efficiencies above 80%, primarily for spiked PS and PMMA NPs, while the general applicability has not yet been investigated with environmental water samples, and the functionalization has relied solely on chemical and physical means without incorporating biological molecules such as material-binding peptides (MBPs).

Analytical methods are essential for the chemical identification and quantification of MP/NPs. Vibrational spectroscopy techniques such as Fourier-transform infrared spectroscopy (FTIR) and Raman spectroscopy can only detect MPs larger than 1 µm^50^, while light scattering methodologies such as Nanoparticle tracking analysis (NTA) enable quantification but cannot distinguish between inorganic particles and organic MP/NPs^51^. Py-GC/MS is a promising methodology, not limited by particle size, that can determine and quantify mixed plastics^52^ and additives^53^. In detail, studies report a successful Py-GC/MS application for detecting and quantifying mixed MP/NPs in environmental and biological samples^27, 35–38, 54–56^, with detection limits, down to 0.04 µg for PP and 0.07 µg for PE^37^.

Fluorescence would also be a highly sensitive quantification methodology if material-specific or general labeling of MP/NPs could be achieved. A proof of concept with Alexa-fluorophore conjugated to antibody-like peptides was reported with a flow cytometry sorting^57^. A general applicability would require a broad array of material-specific binding peptides^58, 59^ or a general peptide binder that can distinguish between inorganic particles and MP/NPs^57^. The MBP technology was summarized including limitations and application potential in a recent comprehensive review^60^, in which the MBPs were divided into naturally occurring binding peptides (nMBPs) and man-made or engineered binding peptides (eMBPs). MBPs are typically smaller than 100 amino acids^60^, and can strongly bind on the surfaces of natural materials, such as leaves^61–63^, graphite^64^, and metals^65, 66^, as well as synthetic materials such as PP^67^, PS^68^, PET^69^, PLA^58, 59^, nylon 66^70^, and stainless steel^71, 72^. A well-studied peptide is the liquid chromatography peak I (LCI) peptide, which consists of 47 amino acids and adopts a β-sheet structure^73^. MBP-1 is an interesting MBP since it is composed of two parallel α-helices and contains arginine in one-third of its 33 amino acids^74^. Interestingly, the incorporation of positively charged amino acids, such as arginine or lysine, has been reported to significantly improve binding strength to polymer surfaces such as PP^75^, PET^70^, and polyamide^70^. MBP-binding generally occurs under ambient temperatures in aqueous systems within a few minutes and could even withstand laundry at 60 °C^70, 76^. Functionalization of SPIONs with MBPs has not yet been reported nor has an application in MP/NPs enrichment. In detail, bifunctional peptides with a first binding domain for SPIONs and a second binding domain to catch the MP/NP particle have not been reported. Bifunctional peptides are usually generated by gene fusion of two MBPs with a stiff linker in between, that spatially separates both domains and thereby ensures their binding functionalites^61, 77^. Reported applications of bifunctional proteins comprise mainly plant health^61–63^, biocatalysis^64, 71, 78^, and medicine^77^.

**Fig. 1:**
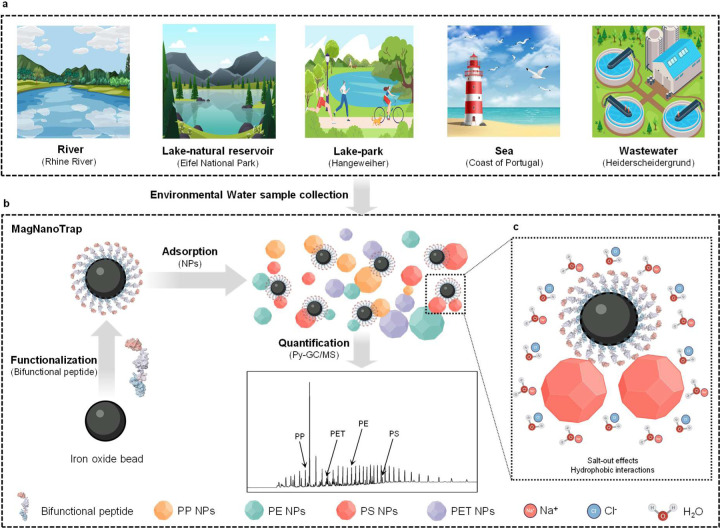
Overview of MagNanoTrap monitoring platform for NPs enrichment and quantification in environmental water samples. **a**, Origins of environmental water samples investigated in this study. **b**, Scheme of the MagNanoTrap enrichment platform to determine and quantify NPs contaminations in water samples with Py-GC/MS. In step 1, SPIONs were decorated with LCI-DZ-MBP1 bifunctional peptide; in step 2, NPs (PP, PE, PS, and PET) were caught by MagNanoTrap beads; and in step 3, NPs composition and quantity were determined by Py-GC/MS. **c**, Scheme of salt effect to facilitate the capture of NPs with MagNanoTrap beads. Protein models are visualized and coloured by ChimeraX 1.4^79^.

Here, we developed and validated the MagNanoTrap termed enrichment platform based on a bifunctional peptide (LCI-DZ-MBP1) combined with SPIONs (Fig. 1). The bifunctional peptide was designed to decorate SPIONs for NPs capture, with LCI binding to SPIONs and MBP-1 acting as a general binder for PP, PE, PS and PET. System validation included evaluating enrichment performance in 200 µL of spiked NPs deionized water, assessing the salt effect on PS NPs adsorption, and testing enrichment performance in 1 L of spiked NPs. MagNanoTrap enabled, in combination with the Py-GC/MS method, to determine the composition of mixed NPs reliably and quantify NP amounts down to 0.061 µg for PS. The achieved sensitivity ensured that a 1 L water sample was often sufficient to detect MP/NP contamination in environmental water samples. The platform demonstrated broad applicability for various polymers, including PP, PE, PS, PET, PMMA, PC, Nylon 6, and Nylon 66, and was successfully tested on environmental water samples from rivers, lakes, seas, and wastewater sources.

## Results

In order to develop and validate the MagNanoTrap monitoring platform for NP-particles, the following steps were carried out to characterize and validate the MagNanoTrap enrichment platform: (A) SPIONs functionalization including the identification of MBPs that bind to SPIONs, and PP-, PE-, PS-, and PET-particles, (B) Enrichment of PS NPs to determine key performance parameters, such as iron oxide concentration, peptide concentration, and peptide functionalization time, (C) Determination of NaCl concentrations and cations effect on enrichment performance, (D) Determination of the recovery obtainable with MagNanoTrap platform for 1 L water samples that are spiked with PP-, PE-, PS-, and PET-particles and subjected to Py-GC/MS analytics, and finally (E) Validation of the MagNanoTrap monitoring platform for quantification of NPs in environmental water samples with Py-GC/MS analysis.

### SPIONs functionalization

**Fig. 2:**
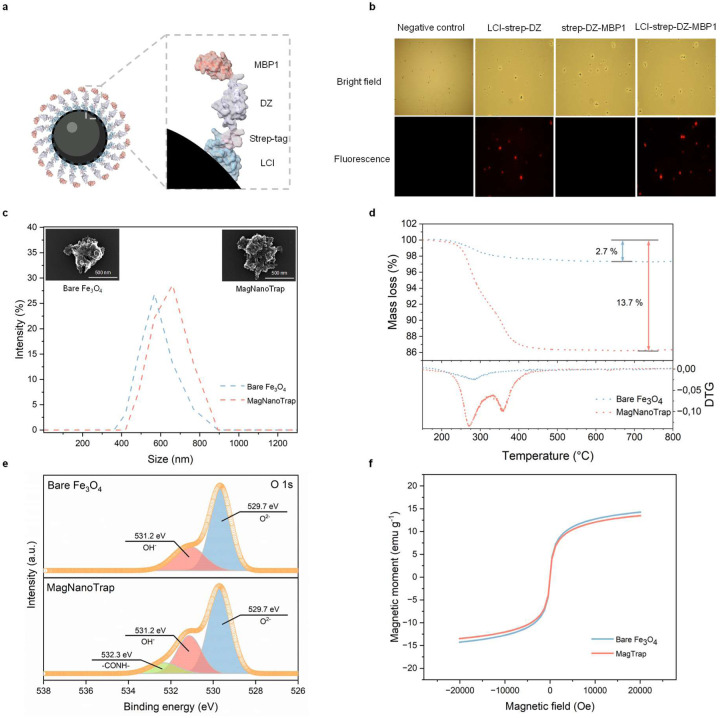
SPIONs functionalization with the bifunctional peptide LCI-strep-DZ-MBP1. **a**, Scheme illustrating decoration of a SPION by the bifunctional peptide LCI-strep-DZ-MBP1. **b**, Experimental data demonstrating the functionalization of SPIONs with single peptides (LCI-strep-DZ and strep-DZ-MBP1) and the bifunctional peptide LCI-strep-DZ-MBP1, visualized through fluorescence microscopy via fluorescent streptavidin binding to the strep-tag (bottom) and microscopy in bright field mode (top). **c**, DLS measurements to determine the size distribution of bare (blue) and functionalized (orange) SPIONs with an average size of 550 nm and 680 nm, respectively. The left and right top SEM images show the morphology of bare and bifunctional peptide-decorated SPIONs. **d**, TGA measurements of bare SPIONs (blue) and MagNanoTrap beads (orange) confirming peptide decoration of SPIONs due to a significantly reduced mass after heating (13.7 % in MagNanoTrap compared to 2.7 % in bare SPIONs). **e**, XPS spectra (O 1s) of bare SPIONs (top) and MagNanoTrap (bottom) confirming the functionalization by the appearance of a peak at 532.3 eV, which is attributed to the -CONH- bonds in proteins. **f**, Magnetization of bare SPIONs (blue) and MagNanoTrap (orange) through vibrating sample magnetometer showing that the thin bifunctional peptide layer does not influence the magnetic properties. An LCI-strep-DZ-MBP1 peptide has a height of 8.5 nm (determined by PyMOL). Protein models are visualized and coloured by ChimeraX 1.4^79^.

Fig. 2 shows the successful decoration of SPIONs (Fe_3_O_4_) with the bifunctional peptide LCI-DZ-MBP1, along with the characterization of the physical properties of both bare SPIONs and peptide-decorated SPIONs. The SPIONs decorated with bifunctional peptides will subsequently be referred to as MagNanoTrap.

Bifunctional peptide decoration of SPIONs was simply achieved by suspending SPIONs in a bifunctional peptide solution at room temperature, followed by a simple washing step with water after a 1-min incubation (Fig. 2a). The binding was demonstrated by employing a fluorescence-labeled streptavidin protein, which specifically binds to the strep-tag present in the fusion proteins LCI-strep-DZ, strep-DZ-MBP1, and LCI-strep-DZ-MBP1. Fluorescent labeling enables visualization of the binding properties on both the SPION surface and the plastic surface. LCI, in the LCI-strep-DZ fusion protein, binds to SPIONs, whereas MBP1 (strep-DZ-MBP1) did not yield a detectable binding signal (Fig. 2b). Further binding studies revealed that MBP1 is a strong binder to a wide range of synthetic polymers, including PP, PE, PS, and PET (Supplementary Fig. 1). The latter results show that the combination of LCI and MBP1 is an excellent bifunctional peptide design for MagNanoTrap development. Additionally, flow cytometry analysis confirmed that 98% of SPIONs were successfully decorated with the bifunctional peptide LCI-DZ-MBP1 (Supplementary Fig. 2). In Fig. 2c, the morphology and size dispersity were determined by scanning electron microscopy (SEM) and dynamic light scattering (DLS). DLS analysis confirmed that no aggregation of MagNanoTrap beads occurred and, as expected, showed an increased hydrodynamic radius (Fig. 2c). SEM images showed that SPION morphology was preserved as expected by decoration with a nanometer-sized peptide layer (Fig. 2c). Thermogravimetric analysis (TGA) confirmed LCI-DZ-MBP1 decoration of SPIONs, with one more peak observed in the derivative curve (Fig. 2d). The presence of peptides on the surface of SPIONs was further confirmed by electrospray ionization coupled with mass spectroscopy (ESI-MS), in which the detected molecular weight closely aligns to the size (17.49 kDa) determined by SDS-PAGE analysis (Supplementary Fig. 3 and Fig. 4). X-ray photoelectron spectroscopy (XPS) also confirmed the successful binding of LCI-DZ-MBP1 to SPIONs. In detail, the O 1s spectrum exhibited a new peak at 532.3 eV for MagNanoTrap when compared to bare SPIONs, which is characteristic of peptide bonds (-CONH-; Fig. 2e). FTIR further confirmed the characteristic of N-H bond (1632 cm^−1^) that LCI-DZ-MBP1 decorates SPIONs (Supplementary Fig. 5). The vibrating sample magnetometer (VSM) showed that the magnetic moment and superparamagnetic behavior of the SPIONs are neglectably influenced by LCI-DZ-MBP1 decoration (Fig. 2f, Supplementary Fig. 6).

The aforementioned characterization indicates a successful decoration of SPIONs with the bifunctional peptide LCI-DZ-MBP1, resulting in the production of MagNanoTrap beads, which will be utilized in subsequent steps for the enrichment of NPs in water samples.

### Enrichment performance

**Fig. 3:**
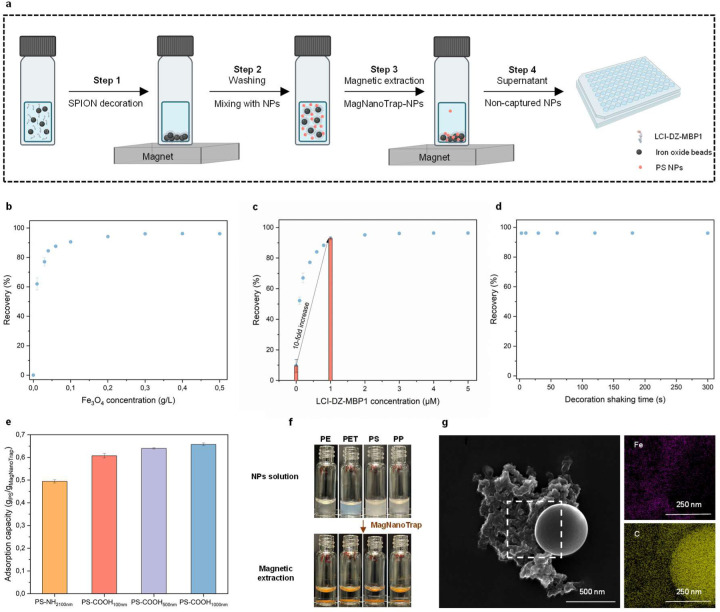
Adsorption efficiency of MagNanoTrap for NPs. **a**, Scheme of the four-step workflow developed for assessing the enrichment performance of MagNanoTrap technology on a 200 µL scale. In step 1, SPIONs were decorated with LCI-DZ-MBP1 in 50 mM Bicine, pH 9; in step 2, the MagNanoTrap beads were washed with water, followed by mixing with NPs in the presence of 150 mM NaCl; in step 3, MagNanoTrap-NPs complex was extracted by a magnet; and in step 4, the supernatant containing non-captured NPs was taken for absorbance measurement. The recovery of PS-COOH _500 nm_ was determined at **b,** increasing MagNanoTrap concentrations, **c**, increasing LCI-DZ-MBP1 peptide concentrations, and **d**, different incubation times of SPIONs and LCI-DZ-MBP1 peptide for SPIONs functionalization. **e**, Adsorption capacity of positively (PS-NH_2 100 nm_) and negatively (PS-COOH _100 nm_, PS-COOH _500 nm_, and PS-COOH _1000 nm_) charged NPs with MagNanoTrap. **f**, Visualization of 0.2 g/L dispersion of PE, PET, PS, and PP NPs before (top) and after magnetic extraction (bottom) by MagNanoTrap beads. **g**, SEM coupled with EDXS images showing PS-COOH _500 nm_ NP was captured by MagNanoTrap beads, in which purple represents Fe elements in SPIONs (top right) and yellow represents C elements in PS NPs and MagNanoTrap beads (bottom right).

Enrichment experiments with PS, PP, PE, and PET NPs were performed on a 200 µL scale to evaluate the performance and general applicability of the MagNanoTrap enrichment technology. Fig. 3a shows the four-step workflow developed for assessing the enrichment performance of scalable MagNanoTrap technology, incorporating absorbance measurement for quantification of enrichment performance (Supplementary Table 1). Enrichment optimization comprised determining the optimal SPION concentration (Fig. 3b), the required LCI-DZ-MBP1 concentration for SPION decoration (Fig. 3c), the minimal incubation time for efficient LCI-DZ-MBP1 decoration of SPIONs (Fig. 3d), and the ideal NaCl concentration (Supplementary Fig. 6). In all experiments, 5 µM of LCI-DZ-MBP1 and 1 min decoration time were used to ensure a saturated decoration of SPIONs, while a 20-min shaking time was employed for saturated PS-COOH _500 nm_ NPs capture, with 150 mM NaCl supplemented in Step 2, unless otherwise specified. In detail, a concentration of 0.3 g/L of decorated SPIONs effectively recovered 0.2 g/L of PS-COOH _500 nm_ NPs, achieving a recovery of 96.1 ± 0.3 % (Fig. 3b). Additionally, 1 µM of peptide was adequate to fully decorate 0.3 g/L of SPIONs, with LCI-DZ-MBP1 functionalization significantly enhancing NPs capture, increasing the recovery ratio by 10-fold compared to bare SPIONs (Fig. 3c). Notably, as shown in Fig. 3d, just 3 seconds of shaking were sufficient for SPION decoration. Interestingly, the enrichment only occurred with the addition of NaCl, with 150 mM of NaCl reaching the saturated recovery (Supplementary Fig. 7).

The general applicability was investigated by applying the developed MagNanoTrap enrichment platform to PS NPs functionalized with amino groups (PS-NH_2_, pKa 9–10) and carboxylate groups (PS-COOH, pKa 4.75). Experiments were performed at pH 7, where PS-NH_2_ NPs are positively charged, while PS-COOH NPs are negatively charged (Supplementary Fig. 7). The adsorption capacities were determined as 494.3 ± 7.0 mg/g for PS-NH_2 100 nm_, 607.6 ± 9.5 mg/g for PS-COOH _100 nm_, 639.8 ± 3.2 mg/g for PS-COOH _500 nm_, and 657.5 ± 6.0 mg/g for PS-COOH _1000 nm_ (Fig. 3e). A general trend was observed, where increasing PS-COOH NP size (100 nm, 500 nm, and 1000 nm) resulted in higher adsorption capacities. Further validation of the enrichment efficiency was performed using three additional NP types: PP, PE, and PET. These self-made NPs, prepared by emulsification-solvent evaporation^80^ and nanoprecipitation^81^ methods, were characterized as negatively charged based on zeta potential measurements. The size distribution and morphology were analyzed using DLS and SEM, respectively, showing that the self-made PP, PE, and PET particles are in nano-size (Supplementary Fig. 8). After mixing with MagNanoTrap beads and performing magnetic extraction, the supernatant of all tested NP suspensions became completely clear, confirming successful NP removal (Fig. 3f, Supplementary Video). Additionally, SEM/EDXS images of MagNanoTrap after capturing PS-COOH _500 nm_ NPs provided further evidence of successful SPION functionalization and NPs adsorption (Fig. 3g). These results collectively validate the bio-functionalized MagNanoTrap platform as a universal NPs enrichment platform.

### NaCl concentration and cations effect

**Fig. 4:**
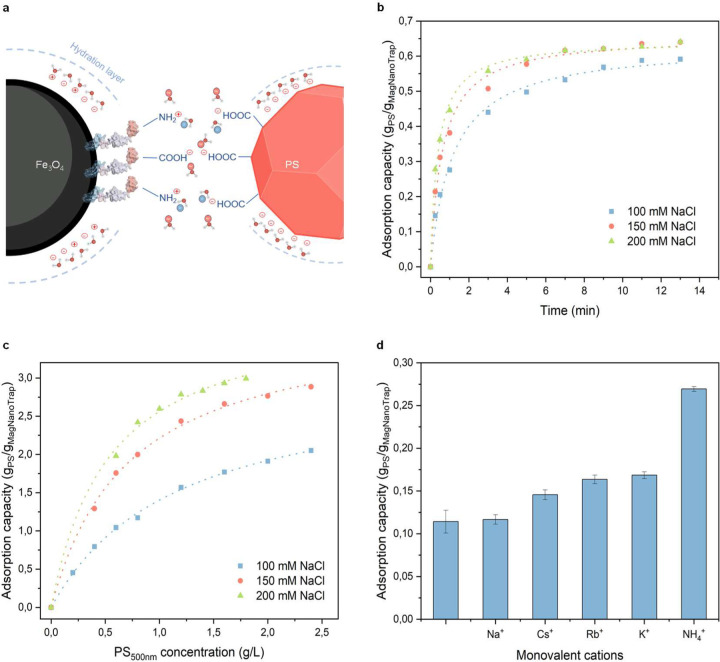
NaCl concentration and cation effects on NPs adsorption. **a**, Scheme illustrating the NaCl effects on NPs adsorption by MagNanoTrap beads. **b**, Adsorption kinetics of MagNanoTrap platform for PS-COOH _500 nm_ NPs at different salt concentrations (100, 150, and 200 mM) fitted with pseudo-second-order model. **c**, Adsorption isotherm of MagNanoTrap platform for PS-COOH _500 nm_ NPs at different salt concentrations (100, 150, and 200 mM) fitted with Langmuir model. **d**, Adsorption capacity of PS-COOH _500 nm_ NPs by MagNanoTrap platform in the presence of 50 mM monovalent cations (Li^+^, Na^+^, Cs^+^, Rb^+^, K^+^, and NH_4_^+^) during a 30-second reaction. Protein models are visualized and coloured by ChimeraX 1.4^79^.

Salt concentrations are known to influence the binding properties of peptides to hydrophobic interaction chromatography (HIC)^82^. In order to develop a generally applicable MagNanoTrap enrichment technology, the effect of salt on the binding properties of the exposed peptide MBP1 to PS NPs was investigated. Salt NaCl was selected to ensure that the MagNanoTrap platform remains functional in seawater environments. During the adsorption process, NaCl ions likely competed with the interactions between water molecules and the charged groups on the surfaces of MagNanoTrap and NPs, weakening the hydration layer, which in turn made the hydrophobic regions more exposed^83^ (Fig. 4a). To gain deeper insight into the role of NaCl in NPs enrichment, NaCl effects on adsorption kinetics and adsorption isotherm were investigated. Kinetic studies showed rapid adsorption of 0.2 g/L PS-COOH _500 nm_ NPs by 0.3 g/L of MagNanoTrap beads, achieving adsorption saturation within 13 min reaction (Fig. 4b). The experimental data aligned with a pseudo-second-order adsorption kinetic model^84^, with the adsorption rate constant, *k*_2_, increasing significantly as the salt concentration rose. Specifically, *k*_2_ values increased from 1.36 ± 0.16 g/(g·min) at 100 mM to 4.04 ± 0.29 g/(g·min) at 200 mM, demonstrating the critical role of NaCl in accelerating the reaction rate. As expected, the adsorption uptake capacity at equilibrium, *q*_e_, remained relatively consistent, with values of 0.63 ± 0.01 g/g, 0.66 ± 0.01 g/g, and 0.64 ± 0.01 g/g for 100 mM, 150 mM, and 200 mM NaCl, respectively (Supplementary Table 2). To determine how the adsorption affinity and maximum adsorption capacity of MagNanoTrap against PS-COOH _500 nm_ NPs is affected by NaCl concentration, the adsorption isotherm was investigated and fitted to both the Langmuir and Freundlich models^85^ (Fig. 4c, Supplementary Table 3). The adsorption data showed a good fit to the Langmuir model, with a correlation coefficient > 0.99, suggesting monolayer adsorption and a uniform adsorption surface of the material^85^. The increase of Langmuir constant, *K*_L_, with NaCl concentration, from 0.85 ± 0.05 L/g at 100 mM to 1.85 ± 0.21 L/g at 200 mM, implied enhanced adsorption affinity due to the presence of NaCl. The maximum adsorption capacities, *q*_m_, calculated by the model were 3.05 ± 0.08 g/g, 3.80 ± 0.09 g/g, and 3.95 ± 0.14 g/g at 100 mM, 150 mM, and 200 mM of NaCl, respectively, outperforming many previously reported materials^86^. Interestingly, at the same NaCl concentration, the adsorption capacity was lower for smaller PS-COOH NPs compared to larger NPs, showing adsorption of 517.6 ± 10.6 mg/g for PS-COOH _100 nm_, 591.2 ± 2.2 mg/g for PS-COOH _500 nm_, and 649.4 ± 5.2 mg/g for PS-COOH _500 nm_ at 100 mM NaCl, since smaller NPs with larger surface area require more NaCl to neutralize the surface charges (Supplementary Fig. 9). To further validate the hypothesis, the enrichment with 50 mM of various monovalent cations was performed over a 30-second reaction. The adsorption capacity of MagNanoTrap mediated by different cations followed the order NH_4_^+^ > K^+^ > Rb^+^ > Cs^+^ > Na^+^ > Li^+^, consistent with the Hofmeister series, which illustrated the salting-out ability of the cations^87^ (Fig. 4d).

In order to assess the applicability of the MagNanoTrap enrichment technology in environmental water samples containing heavy metals, enrichment experiments were performed using Cd^2+^, Zn^2+^, Cu^2+^, and Ni^2+^, instead of NaCl. The results demonstrated a recovery exceeding 99% for PS-COOH _500 nm_ NPs, confirming that the presence of heavy metals does not interfere with the enrichment process (Supplementary Fig. 10). The heavy metal ions might serve as bridges between the MagNanoTrap beads and NPs during the enrichment process via Lewis acid-base interaction^88^ (Supplementary Fig. 11). Using Zn^2+^ as a model, the adsorption capabilities of MagNanoTrap beads and PS-COOH _500 nm_ NPs were evaluated, both individually and in combination, for adsorbing heavy metals. At a Zn^2+^ concentration of 20 mM, MagNanoTrap beads (0.3 g/L) showed an uptake of 1292.3 ± 55.5 µM of Zn^2+^, while PS-COOH _500 nm_ NPs (0.2 g/L) alone, adsorbed 538.5 ± 40.7 µM of Zn^2+^. Interestingly, when PS-COOH _500 nm_ NPs were incubated in combination with MagNanoTrap beads, the total Zn^2+^ adsorption reached only 1456.4 ± 38.7 µM, less than the sum of the individual adsorption capacities, suggesting that Zn^2+^ ions mediate the interaction between PS-COOH _500 nm_ NPs and MagNanoTrap beads (Supplementary Fig. 12).

The aforementioned results showed that NaCl supplementation enhances MBP1 binding to plastic surfaces, and heavy metals do not influence the enrichment process. It highlights the general applicability of the MagNanoTrap platform, which can be utilized in all kinds of natural aqueous environments, such as lakes, rivers, wastewater, and seawaters, as NaCl can always be added.

### Enrichment performance on a 1 L scale characterized by Py-GC/MS

**Fig. 5:**
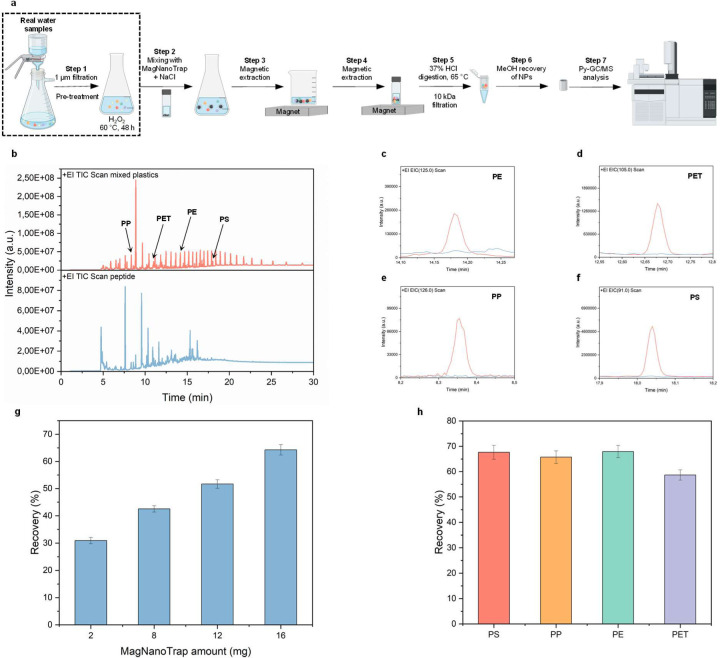
1 L-scale enrichment characterized by Py-GC/MS. **a**, Scheme of the seven-step workflow developed for assessing the enrichment performance of MagNanoTrap technology on a 1 L scale. In step 1, 1 µm filtration and H_2_O_2_ digestion were done to remove the large aggregates and organic matter; in step 2, MagNanoTrap beads were added to the pretreated environmental water samples with the supplementation of NaCl; in steps 3 and 4, MagNanoTrap-NPs complex was extracted by a magnet to reduce the volume; in step 5, MagNanoTrap-NPs complex was digested by 37% HCl at 65 °C, followed by filtration with 10 kDa centrifugal filters; in step 6, the complex was recovered with methanol and transferred to Py-GC/MS sampling cups; and in step 7, NP quantification was done by Py-GC/MS. **b**, Chromatograms of the mixed plastics containing PP, PE, PET, PS, PMMA, PC, PVC, nylon 6, nylon 66, ABS, and SBR (top) and the peptide LCI-DZ-MBP1 (bottom). Chromatograms with an extracted m/z of, **c**, PE (m/z 125), **d**, PET (m/z 105), **e**, PP (m/z 126), and **f**, PS (m/z 91) from both mixed plastics and peptide, showing no interference by the peptide LCI-DZ-MBP1 at the respective peaks. **g**, Recovery of PS-COOH _500 nm_ NPs (5 µg spiked in 1 L deionized water) at different amounts of MagNanoTrap beads, with 1 M NaCl and 2 h shaking. **h**, Recovery of mixed NPs (PS, PP, PE, and PET, each spiked with 1 µg in 1 L deionized water) by 16 mg of MagNanoTrap beads.

In this section, we aim to prove that the NaCl-facilitated enrichment protocol can be scaled up to a 1 L volume by investigating the recovery of spiked mixed PP, PE, PET, and PS NPs in deionized water. Fig. 5a shows the seven-step workflow developed for assessing the enrichment performance of MagNanoTrap technology on a 1 L scale, incorporating Py-GC/MS analysis for the detection and quantification of mixed plastics. To ensure accurate plastic quantification, extracted peaks from the mixed plastics, including PP, PE, PET, PS, PMMA, PC, PVC, nylon 6, nylon 66, ABS, and SBR were compared against those from the bifunctional peptide LCI-DZ-MBP1 (Fig. 5b). The specific molecular markers used for PP, PE, PET, and PS quantification were 2,4,6-dimethyl-1-heptene (m/z 126), 1-heptadecene (C17, m/z 125), monomethyl terephthalate (m/z 105), and styrene trimer (m/z 91), respectively^36, 37, 89^. Notably, at the respective m/z values, signals were only observed from plastics, confirming no interference from the peptide in the identification of PE (Fig. 5c), PET (Fig. 5d), PP (Fig. 5e), and PS (Fig. 5f) NPs. To prevent interference from iron oxide in NP quantification, the recovered MagNanoTrap-NP complex was treated with 37% HCl at 65 °C, leading to iron oxide digestion and aggregate (peptide-NPs complex) formation, prior to Py-GC/MS analysis (Supplementary Fig. 14).

To evaluate the 1 L-scale enrichment performance of MagNanoTrap for trace amounts of PS-COOH _500 nm_ NPs, optimization comprised determining the ideal quantity of MagNanoTrap beads (Fig. 5g), the required incubation time for PS-COOH _500 nm_ NPs recovery (Supplementary Fig. 15), and the optimal NaCl concentration (Supplementary Fig. 16). Specifically, for 5 µg of spiked PS-COOH _500 nm_ NPs, an increasing amount of MagNanoTrap beads resulted in improved recovery, with 16 mg of beads achieving a 64.3% recovery (Fig. 5g). The general applicability was further investigated by enriching a mixed NP suspension containing 1 µg each of PP, PE, PET, and PS, yielding recovery of 65.8%, 68.0%, 67.7%, and 58.7%, respectively (Fig. 5h). In summary, these results validate the scale-up to 1 L samples with the employed concentrations down to 1 µg/L, making the MagNanoTrap enrichment technology an attractive tool for NP quantification in natural environments.

### NPs quantification in 1 L environmental water samples

**Fig. 6:**
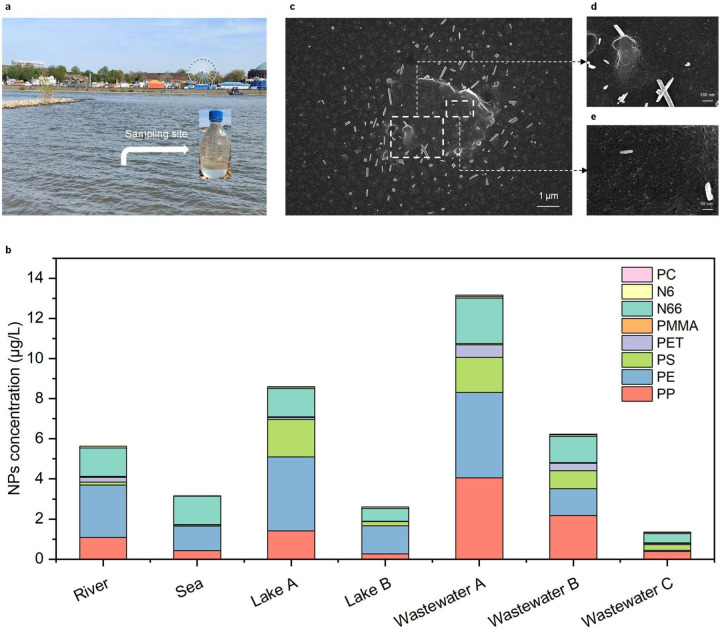
NPs quantification from environmental water samples. **a**, Representative sampling site of the river sample in the Rhine River, Düsseldorf, Germany. **b**, Quantification and determination of plastic composition in seven types of environmental water samples: river water from the Rhine River, seawater from the coast of Portugal, lake water from the Hangeweiher Park (Lake A) with high human activity, lake water from the Eifel natural reservoir (Lake B) with restricted human activity, wastewater samples obtained from the effluent of the wastewater treatment plant Heiderscheidergrund (Wastewater A), wastewater after sand filtration (Wastewater B) and wastewater after ultrafiltration (Wastewater C). **c**, SEM images showing the morphology and size distribution of enriched NPs by MagNanoTrap from wastewater after ultrafiltration (Wastewater C), with insets displaying zoomed areas of NPs **d**, in 100 nm bar and **e**, 50 nm bar.

The application of the MagNanoTrap enrichment technology to quantify NP contaminations in environmental water samples was probed by investigation of seven environmental water samples, including freshwater from the Rhine River (Fig. 6a), seawater from the coast of Portugal, lake water from the Hangeweiher Park with high human occurrence, lake water from the Eifel natural reservoir with low human activity, and wastewater from the effluent of the Heiderscheidergrund wastewater treatment plant (WWT). Wastewater samples were also collected after sand filtration and ultrafiltration to determine the effects of advanced treatment processes in WWTs. The selected samples cover fresh water, salt water, and wastewater to determine the application scope of the MagNanoTrap enrichment technology. All environmental water samples were pretreated with H_2_O_2_ to effectively remove organic matter from the NPs' surfaces, ensuring MBP1 binding and accurate quantification by Py-GCMS^37, 38^ (Fig. 5a).

In order to determine whether the recovery efficiency is affected by components within the environmental water samples, a set of spiked experiments was performed with all seven environmental water samples by spiking them with PP, PE, PS, and PET NPs (1 μg/L each). The recoveries, ranging from 51.9% to 60.6% for all environmental samples (Supplementary Table 6), compared to 58.7% to 68.0% in deionized water, highlight the robustness of the MagNanoTrap platform for use in diverse environmental water conditions. With no peak interference from the peptide (Supplementary Fig. 17), four additional types of plastics were also successfully detected and quantified, including PMMA, Nylon 6, Nylon 66, and PC. The detected concentrations of NPs in river water, seawater, lake water in the park, lake water in the natural reservoir, wastewater from effluent, wastewater after sand filtration, and wastewater after ultrafiltration were 5.64 µg/L, 3.16 µg/L, 8.60 µg/L, 2.61 µg/L, 13.16 µg/L, 6.23 µg/L, and 1.35 µg/L, respectively (Fig. 6b). Interestingly, lake water from the park, influenced by human activities, contained 3.3 times more NPs than lake water from the natural reservoir. For wastewater after different treatments, the plastic content significantly decreased, with ultrafiltration membrane pretreatment removing up to 90% of NPs (Fig. 6b). Remarkably, PP, PE, and Nylon 6,6, the dominant NP types detected in the water samples, accounted for over 75% of the total plastic content (Supplementary Fig. 18). The SEM analysis revealed that the enriched samples contained irregular morphologies of NPs, such as fiber-like, flake-like, and ball-stick forms, particularly from wastewater after ultrafiltration, with sizes below 100 nm (Fig. 6c, Supplementary Fig. 19).

## Discussion

A circular polymer economy requires that MP/NP-particle contamination is monitored to ensure environmental, animal, and human health. The EU Commission has recognized the absence of reliable analytical methods for detecting, quantifying, and removing MP/NPs as a major barrier to regulating MP/NP contamination^30, 31^, emphasizing the need for universally applicable enrichment and analytical technologies.

We developed a biofunctionalized MagNanoTrap platform that is readily prepared and universally applicable for NPs enrichment. Here, SPIONs were first combined with a bifunctional peptide, LCI-DZ-MBP1, giving an innovative solution for water remediation. Most existing MP/NP removal/enrichment techniques rely on recovering commercially available plastic particles, like PS and PMMA, spiked in deionized water, without evaluating their performance in real environmental water samples. The MagNanoTrap platform assessed in spiked assays at a microliter-scale, achieved over 96% recovery for PS, PP, PE, and PET NPs, with a maximum adsorption capacity ranging from 3.05 ± 0.08 g/g to 3.95 ± 0.14 g/g under varying NaCl concentrations, outperforming previously reported adsorbent materials, such as biomass fibrous foam (444.6 ± 22.3 mg/g)^40^ and SPIONs-PAC_18_ (0.69 ± 0.26 g/g)^48^ for PS NPs. The adsorption process is facilitated by salt ions, which eliminate interactions between water molecules and the charged groups on the protein and NP surfaces, weakening the hydration layer and making hydrophobic interactions dominant^83^. The elevated salt concentration utilized for enrichment enhances the system versatility, making it effective even in high-salinity environments like seawater.

In environmental water samples, universally applicable methods for detecting and quantifying mixed plastics have only been reported using membrane-based techniques^35–38^. However, due to membrane cutoff limitations, smaller NPs often stay undetected. In contrast, the MagNanoTrap platform efficiently extracts NPs of all sizes from 1 L environmental water samples (Fig. 6c), achieving comparable recoveries of 56.3 ± 4.3% for PP, PE, PS, and PET NPs. Using the MagNanoTrap platform on seven environmental water samples enabled the detection and quantification of eight types of NPs—PP, PE, PS, PET, PMMA, PC, Ny6, and Ny66—comparable to previously reported methods^35–38^. Notably, lake water from a city park exhibited 3.3 times higher NP contamination than lake water from a nature reservoir, highlighting the impact of human activities. While ultrafiltration treatment in wastewater facilities removes up to 90% of NPs, particles smaller than 100 nm persist and are discharged into domestic water systems. Given their high surface area, these smaller particles have an increased capacity to adsorb toxic compounds^18^.

The combination of bifunctional peptides, SPIONs, and Py-GC/MS analytics has proved to be an excellent analytical method to quantify NP contaminations in environmental water samples as shown for lake water, river water, seawater, and wastewater. One liter of environmental water sample was sufficient to successfully detect and quantify PP, PE, PS, PET, PC, Nylon 6, and Nylon 66 NPs, with detection limits down to 0.81 ng for PC. General applicability for all kinds of man-made polymers (e.g., Teflon and carbon fibers), or other particles (e.g., metals/metal oxides and silica) contaminants is ensured through the modular concept of bifunctional peptides, in which the NP-binding peptide could be replaced by other material-binding peptides^65, 90–92^. It is furthermore very likely that the developed MagNanoTrap monitoring platform can be used in effluents of polymer-producing factories, food samples, or human tissue samples. We hope that in the long run, the MagNanoTrap platform will become a routine method to monitor NP contamination within a circular polymer economy and help researchers and regulatory bodies achieve the goals of SDG6 with respect to clean water^93^.

## Methods

### Materials

Fe_3_O_4_ nanoparticles in water dispersion were purchased from Particular Materials SRL (Italy). PS nanoparticles with different charges and sizes were purchased from Polysciences Europe GmbH (Germany). LD-PE and PET powder were purchased from Goodfellow Cambridge Ltd (Germany). PP granule was purchased from Sigma-Aldrich (Merck, Germany). Strep-Tactin^®^XT DY-649 was purchased from IBA Lifesciences GmbH (Germany). Microplastics calibration standard-low set was purchased from Frontier Lab (Japan). Sodium chloride, hydrogen peroxide, zincon sodium salt, xylene, and other laboratory-grade chemicals were purchased from Sigma-Aldrich (Merck, Germany) or AppliChem (Germany) unless specified.

### Protein expression and purification

The DNA sequences encoding LCI-strep-DZ, strep-DZ-MBP1, and LCI-strep-DZ-MBP1 (Supplementary Table 7) were synthesized and inserted into the pET-28a (+) vector by GenScript Biotech (Netherlands). The plasmids were transformed into competent *E. coli* BL21(DE3) cells for protein expression. Following overnight incubation at 37 °C in a shaker, the pre-culture was used to inoculate 50 mL of Lysogeny Broth (LB) medium (supplemented with 50 µg/mL kanamycin) and cultured at 37 °C with continuous shaking at 200 rpm. When the OD_600_ reached 0.4–0.6, the main cultures were induced with 0.1 mM IPTG, and further incubated at 18 °C, 200 rpm overnight. Cells harvested by centrifugation were resuspended in buffer A (50 mM Tris-HCl, pH 8, and 300 mM NaCl) and lysed by sonication. The supernatant collected after centrifugation was applied to a gravity column packed with Strep-Tactin^®^XT 4Flow^®^ resin, pre-equilibrated with buffer A. After washing with 10 column volumes of buffer A, strep-tagged proteins were eluted with buffer B (50 mM Tris-HCl, pH 8, 300 mM NaCl, and 2.5 mM desthiobiotin). Buffer exchange of the eluted protein into the final stock buffer C (50 mM Bicine-NaOH, pH 9) was performed with a PD-10 Desalting Column (Cytiva). The whole purification and buffer exchange process was done at 4 °C, and the protein concentration was measured by UV absorption at 280 nm.

### Preparation of MagNanoTrap beads

The purified protein was mixed with Fe_3_O_4_ nanoparticles in a functionalization buffer (50 mM Bicine/NaOH, pH 9) by shaking at 1200 rpm in glass vials (Screw neck vial ND 8, TH. GEYER GmbH & Co. KG, Germany). Magnetic extraction was then used to wash the beads with water, after which they were resuspended in water for subsequent use.

### Characterization

The fluorescence microscope (BX51, Olympus) was used to check the binding of peptides on material surfaces. The fluorescence-activated cell sorting (FACS; MoFlo Astrios EQ, Beckman Coulter) was used to analyze the functionalization efficiency of MagNanoTrap beads. The dynamic light scattering (Zetasizer, Malvern Panalytical) was used to measure the size distribution of nanoparticles. The thermogravimetric analysis (TGA; STA6000, PerkinElmer) was used to examine the mass loss of samples during temperature increase. The X-ray photoelectron spectroscopy (XPS; AXIS supra +, Kratos Analytical) and Fourier-transform infrared spectroscopy (FTIR; Nicolet iS20, Thermo Scientific) were used to study the surface chemistry of magnetic beads. An electrospray ionization time-of-flight mass spectrometry (ESI-TOF MS; Agilent) was used to assess the successful functionalization of magnetic beads by bifunctional peptide. The SQUID magnetometer (MPMS-XL, Quantum Design) was used to study the magnetic properties of the beads. A scanning electron microscope (SEM; SU9000, Hitachi) was to study the morphology of the samples. A transmission electron microscopy (TEM; SU9000, Hitachi) was used to observe the inner structure of particles.

### Preparation of PP and PE nanoplastics

PP and PE nanoparticles were synthesized as described by Faeze *et al.*^80^ with modifications. Briefly, 10 mg of PP granules or LD-PE powder were dissolved in 5 mL of xylene at 110 °C in a glass beaker on a heater for 10 minutes until the solids were completely dissolved. With vigorous stirring, 20 mL of icy deionized water was rapidly added to emulsify the mixture, followed by immediate water bath sonication for 30 minutes. Once the PP/PE particles were suspended in water, Whatman Grade 1 qualitative filter paper was used to filter out the larger particles. The filtration process was repeated one more time after mild magnetic stirring for 2 hours at room temperature to obtain the nanoparticle dispersion in water.

### Preparation of PET nanoplastics

PET nanoparticles were prepared according to a reported protocol^81^. Briefly, 10 mg of PET powder was dissolved in 2 mL 1,1,1,3,3,3-hexafluoro-2-propanol at room temperature for 1 h. The PET solution was dropped into 20 mL of deionized water with vigorous magnetic stirring. The organic solvent was removed from the particle suspension by continuous mild stirring for 2 hours at room temperature, followed by paper filtration to separate the precipitated particles.

### 200 µL-scale Enrichment

Enrichment on a 200 µL scale was done in the glass vial and characterized by UV absorbance measurement (CLARIOstar, BMG Labtech). Specifically, the prepared MagNanoTrap beads were mixed with 0.2 g/L of PS NPs and 150 mM NaCl in a water solution at a total volume of 200 µL. Followed by shaking at 1200 rpm for 20 min and magnetic extraction, 100 µL of supernatant was transferred into a 96-well plate for absorbance characterization. The concentration was obtained according to the UV-vis standard curves and the NPs recovery rate $\left(R_{1}\right)$ was calculated using equation (1),

(1) $$R_{1}=\left(1-\frac{C_{t}}{C_{0}}\right)*100\%$$

where $C_{t}$ represents the NPs concentration in the supernatant after magnetic extraction and $C_{0}$ is the concentration of the original NPs water dispersion. The adsorption uptake $q\left(g/g\right)$ was calculated using equation (2),

(2) $$q=\frac{C_{0}-C_{t}}{C_{m}}$$

where $C_{m}$ is the concentration of MagNanoTrap beads. Each experiment was done in triplicates.

### Kinetic and isotherm models

The adsorption kinetic process was described by two adsorption models in this study, including Pseudo-first-order (3) and Pseudo-second-order (4)^84^.


(3) $$q_{t}=q_{e}\left(1-e^{-k_{1}t}\right)$$

(4) $$q_{t}=\frac{k_{2}q_{e}^{2}t}{1+k_{2}q_{e}t}$$

The $q_{t}$ and $q_{e}$ are the amounts of NPs adsorbed on per unit mass of MagNanoTrap beads at time, *t* and at equilibrium, respectively; $k_{1}$ and $k_{2}$ are adsorption rate constants for the Pseudo-first-order and Pseudo-second-order, respectively.

The adsorption isotherm was described by two adsorption models in this study, including Freundlich model (5) and Langmuir model (6)^85^.


(5) $$q_{e}=K_{F}C_{e}^{1/n}$$

(6) $$q_{e}=\frac{q_{m}K_{L}C_{e}}{1+K_{L}C_{e}}$$

The $K_{F}\left(\text{L/g}\right)$ and $K_{L}\left(\text{L/g}\right)$ are the isotherm constants for Freundlich and Langmuir model, respectively; $C_{\text{e}}\left(\text{g}/\text{L}\right)$ is the concentration of adsorbate, PS NPs; $q_{m}$ and $q_{e}$ are adsorption capacity at maximum and equilibrium, respectively.

### Zn^2+^ adsorption

The concentration of Zn^²⁺^ was measured using a zincon assay, with the absorbance recorded at 620 nm via UV-vis spectrophotometry. Briefly, 0.3 g/L MagNanoTrap beads and 0.2 g/L PS-COOH 500 nm NPs were mixed with 20 mM Zn^²⁺^, either individually or together, and shaken at 1200 rpm for 10 minutes. After centrifugation at 10,000 rpm for 1 minute, the supernatant was diluted 80-fold and mixed with 1 mM zincon solution for concentration analysis.

### Environmental water sample collection

The lake water samples in the natural reservoir were collected from Eifel National Park (Monschau, Germany). The lake water samples from the park with human activities were collected from Hangeweiher (Aachen, Germany). The river water samples were collected from the Rhine River (Düsseldorf, Germany). The seawater from a depth of 50 m off the coast of Portugal was ordered from Mrutzek Meeres-Aquaristik GmbH (Germany). The wastewater was collected from the efflux of the wastewater treatment plant SIDEN located in Heiderscheidergrund (Luxembourg). Wastewater samples from the efflux, after sand filtration and ultrafiltration using a crossflow membrane from Boll & Kirch, were supplied by APATEQ (Luxembourg). All water samples were stored in glass bottles with PP caps at 4 °C, except for the seawater, which was contained in a PE bottle.

### 1 L-scale enrichment

For method optimization, deionized water spiked with NPs was used, whereas environmental water samples required a pretreatment process before extraction. In detail, each 1 L water sample underwent vacuum filtration through 1 µm glass fiber filters (47 mm) on a stainless steel filtration unit to remove large aggregates (Fig. 5a). The filtrate was collected in a 5 L glass flask, followed by oxidative digestion with 100 mL of 30% H₂O₂ at 60 °C for 48 hours in an orbital incubator shaker to remove the organic matter (Minitron, INFORS, Switzerland)^37, 38^. After digestion, the samples were cooled down to room temperature, mixed with 16 mg of MagNanoTrap beads and 1 M NaCl, and shaken at 200 rpm for 2 h to facilitate adsorption. The water hybrid was poured into a 500 mL glass beaker for magnetic extraction of NPs. The extracted mixture was resuspended in 5 mL of deionized water and transferred into a 1.5 mL glass vial for magnetic extraction to further reduce the volume. Subsequently, the MagNanoTrap-NPs complex was treated with 37% HCl at 65 °C for 30 min, followed by filtration using a 10 kDa centrifugal filter (VWR, UK), after which the aggregates were resuspended in methanol and transferred into a Py-GCMS sampling cup for later analysis. The recovery rate (*R*_2_) of the spiked assay was calculated using equation (7),

(7) $$R_{2}=\frac{m_{2}-m_{1}}{m_{0}}*100\%$$

where $m_{0}$, $m_{1}$, and $m_{2}$ were the spiked amounts of NPs, and detected amounts of NPs before and after spiking, respectively.

### Nanoplastics quantification by Py-GCMS

Quantification of eight types of NPs, including PP, PE, PS, PET, Nylon 6, Nylon 66, PC, PMMA, and PVC, was performed using a Multi-Shot Micro-furnace Pyrolyzer (EGA/PY-3030D) with an Auto-Shot Sampler (AS-2020E) from Frontier Laboratories (Japan), coupled to an Agilent GC/MS (8890 GC System and 7000D GC/TQ, USA). The Py-GC/MS analysis parameters were chosen according to previously reported methods (Supplementary Table 4)^35–38^. To identify and quantify plastics in environmental samples, specific indicator compounds/ions are necessary and summarized (Supplementary Table 5). For the calibration curve preparation, varying amounts (1.0, 3.0, 5.0, 7.0, and 9.0 mg) of the standard mixture (MPs-SiO_2_ provided by Frontier Laboratories) were weighed into the pyrolysis cup (80 μL) prior to analysis. The Py-GC/MS method was validated by assessing linearity (R^2^), limit of detection (LOD), and limit of quantification (LOQ), with these parameters calculated for each plastic polymer using the corresponding calibration curves and applying equations (8) and (9), respectively^35–38^:

(8) $$LOD=3x\frac{\alpha }{S}$$

(9) $$LOQ=10x\frac{\alpha }{S}$$

where α represents the residual standard deviation of the regression equation, while S denotes the slope of the calibration curve.

### Nanoplastics visualization by SEM

Following NP enrichment from each environmental water sample in a 1.5 mL glass vial, the samples were treated with 200 µL of 6 M HCl, and shaken at room temperature overnight to dissolve the iron oxide. After acid digestion, the samples were analyzed using SEM.

## Supplementary Material

1

## Data Availability

The paper and/or the supplemental information contain all the data needed to evaluate the conclusions. Source data are provided in this paper.
