# Supplementary material for: MagNanoTrap enrichment empowers ultra-sensitive quantification of mixed nanoplastic particles from environmental water samples
Source: Res Sq. 2025 Jul 14:rs.3.rs-6254645. Preprint. [Version 1] doi: 10.21203/rs.3.rs-6254645/v1 (PMC12288542; doi:10.21203/rs.3.rs-6254645/v1)
Supplement: 1 [file NIHPPrs6254645v1-supplement-1.pdf]

Supplementary Files

This is a list of supplementary files associated with this preprint. Click to download.

- Supplementaryinformation.pdf
- MagneticextractionofPSNPs.mov
